# Soil Microbial Community and Its Interaction with Soil Carbon Dynamics Following a Wetland Drying Process in Mu Us Sandy Land

**DOI:** 10.3390/ijerph17124199

**Published:** 2020-06-12

**Authors:** Huan He, Yixuan Liu, Yue Hu, Mengqi Zhang, Guodong Wang, Weibo Shen

**Affiliations:** 1 College of Science, Northwest Agricultural and Forestry University, Yangling 712100, China; hehuan9009@163.com; 2 College of Natural Resources and Environment, Northwest Agricultural and Forestry University, Yangling 712100, China; liuyx17@lzu.edu.cn (Y.L.); yuehunwafu@163.com (Y.H.); zhangmengqi@nwafu.edu.cn (M.Z.)

**Keywords:** wetland drying, soil microbial biomass, microbial community structure, carbon mineralization, soil-microbial interaction

## Abstract

Increasing drought globally is a severe threat to fragile desert wetland ecosystem. It is of significance to study the effects of wetland drying on microbial regulation of soil carbon (C) in the desert. In this study, we examined the impacts of wetland drying on microbial biomass, microbial community (bacteria, fungi) and microbial activity [basal microbial respiration, microbial metabolic quotient (qCO_2_)]. Relationships of microbial properties with biotic factors [litter, soil organic carbon (SOC), total nitrogen (TN), total phosphorus (TP)], abiotic factors (soil moisture, pH and clay content) and biological processes (basal microbial respiration, qCO_2_) were also developed. Results showed that the drying of wetland led to a decrease of soil microbial biomass carbon (MBC) content, microbial biomass nitrogen (MBN) content and fungi and bacterial abundance, and an increase of the fungi:bacteria ratio. Wetland drying also led to increased soil basal respiration and increased qCO_2_, which was attributed to lower soil clay content and litter N concentration. The MBC:SOC ratios were higher under drier soil conditions than under virgin wetland, which was attributed to stronger C conserve ability of fungi than bacteria. The wetland drying process exacerbated soil C loss by strengthening heterotrophic respiration; however, the exact effects of soil microbial community structure on microbial C mineralization were not clear in this study and need further research.

## 1. Introduction

It is predicted that there will be severe and widespread droughts globally in the next 30–90 years resulting from either decreased precipitation or increased evaporation [1,2,3]. These changes are predicted to exacerbate processes leading to land degradation and desertification and a worldwide decrease in soil moisture by 5–15% has been predicted for the 2080–2099 period [2,4]. The increase of drought will definitely affect the existence and functions of wetlands globally, especially for the wetlands in drylands and desert lands [5,6].

Increasing drought could significantly affect many of the biological and chemical processes in wetland ecosystems [6,7], and the most rapid and prominent change is the modification of microbial community structure and activity [8,9]. Soil microorganisms play a predominant role in regulating the conservation and release of SOC (soil organic carbon) [10]. Soil microorganisms degrade litter and then allocate the carbon to microbial biomass, exudate carbon as microbial derived organic matter or release carbon by heterotrophic respiration [11,12]. Studying microbial biomass, microbial community structure and activity related to C mineralization [soil basal respiration, microbial metabolic quotient (qCO_2_)] are critical for understanding the microbial regulation of SOC stock and release in wetland drying process.

Soil microbial biomass carbon (MBC), microbial biomass nitrogen (MBN) and microbial quotient (MBC:SOC ratio) are important indexes of soil quality and soils with higher MBC and MBN contents and higher MBC:SOC ratios could have stronger ability to conserve SOC [13,14]. In wetland drying process, soil biomass can be affected by changes of soil water content (SWC), physical properties (pH, clay and sand content), nutrient status (nitrogen and phosphorus) and quantity and quality of substrates (soil and litter C:N ratio) [15,16]. It was reported that low SWC and lower amount of SOC and litter could lead to a lower amount of soil MBC and MBN content [17,18]. Previous studies also showed that lower SWC, soil and litter C:N ratios and higher soil total nitrogen (TN) contents could lead to lower MBC:SOC ratios [17,19]. Thus far there has been little research about the responses of soil MBC, MBN and MBC:SOC ratios on the wetland drying process in sandy lands globally.

Microbial community structure was also closely related to SOC reserve and C mineralization in an ecosystem, since soil bacteria and fungi differentially influence the formation and stabilization of different SOM components [11]. Fungi are also known to have slower biomass turnover rates and lower microbial metabolic quotient (qCO_2_) than bacteria [11]. Increasing aridity in wetland ecosystems could affect the soil microbial community directly by decreasing SWC or indirectly by influencing soil pH, clay and sand contents, soil and litter C:N ratios and formation of aggregates [6,20,21]. Previous studies showed that increasing aridity in soil could lead to higher fungal abundance and lower bacterial abundance [22]. Changes in soil moisture can also affect microbial community structure by influencing substrate quality (C:N ratio), and it was reported that higher soil and litter C:N ratios could lead to higher fungi:bacteria ratio [12].

Soil basal respiration and qCO_2_ were closely related to C release in microbial heterotrophic respiration [23,24,25]. Microbial metabolic quotient (qCO_2_) refers to the CO_2_ released per unit of biomass and was a critical indicator of microbial carbon use efficiency [26,27]. Low qCO_2_ values usually indicate higher carbon utilization efficiency and higher stability of soil ecosystem [28]. A previous study showed that changes in soil moisture could affect soil respiration by influencing the aboveground biomass and diversity of vegetation [29]. It was also reported that changes in SWC could affect qCO_2_ directly or indirectly by influencing substrate C:N and C:P ratios and soil physical properties [24,30]. Low SWC and soil clay content and high substrate C:N and C:P ratios could lead to low C use efficiency and high qCO_2_ [24,26,28].

Mu Us sandy land is one of the four biggest sandy lands in China, and is located between Shaanxi province and Inner Mongolia autonomous region. Sandy lands can include all land types covered with sand (including deserts) and can also refer to sandy lands in semi-arid and semi-humid regions. Deserts usually refer to the sandy lands in arid climate region specifically and the major differences between a sandy land and a desert are in precipitation and vegetation [31]. Compared with deserts, sandy lands usually have higher precipitation and higher vegetation coverage. Sandy lands also had more stabilized sand dunes and less mobile dunes because of the existence of vegetation and soil crust [31,32]. Mu Us sandy land is the location of the critical 400 mm precipitation line that separates the semi-humid region and semi-arid region. The ecosystem in this sandy land is quite fragile, which means that the ecosystem has comparatively lower stability and is vulnerable to changes of the environment, such as drought and pollutions of heavy metal or organic pollutants [33]. Wetlands, such as rivers and lakes, play important roles in maintaining the ecosystem and environmental health. However, the increasing drought has led to a shrinkage of water bodies in wetlands, and thus, the ecosystem of wetlands is severely threatened. In previous research, we studied the stock and release of SOC in the wetland drying process in Mu Us sandy land [7]. However, there is still a lack of information on the mechanism and potential of soil C dynamics during the wetland drying process, especially the contribution of soil microbes.

The major objectives of this study were to examine: (1) the impacts of wetland drying on microbial biomass, microbial community (bacteria, fungi) and microbial activity (basal microbial respiration, qCO_2_); and (2) the relationships of microbial properties with biotic factors (litter, SOC, TN, TP), abiotic factors (soil moisture, pH and clay content) and biological processes (basal microbial respiration, qCO_2_).

## 2. Materials and Methods

### 2.1. Study Site and Vegetation Inventory

The location of Mu Us Desert is 107°20′–111°30′ E and 37°27.5′–39°22.5′ N and the sandy land covers northern Shaanxi province and southern Inner Mongolia region. The total area of Mu Us Desert is 42,200 km^2^. The annual temperature of Mu Us Desert is 6.0 to 8.5 °C and the annual precipitation is 150 to 450 mm [34]. For the landscape, stabilized and semi-stabilized sand dunes are widely distributed in the Mu Us sandy land [31]. From west to east and from south to north, the vegetation and soil in the Mu Us Desert are characterized by transitional properties [35,36].

In this study, a lake wetland in the southeastern Mu Us Desert (Jinjie county, China) was selected to conduct the research. The wetland drying process was divided into five different successional stages according to the properties of vegetation and soil water condition. The five successional stages are shown as follows:

1. Stage TAW means virgin wetland stage. Stages TAW has water depths of 70–120 cm and is characterized by *Typha angustifolia*, which is a tall hydrophyte with a height higher than 2 m.
2. Stage PAW is the area near virgin wetland and is the first stage of dry land area. There is no water submerging in this stage, and soil in PAW is comparatively wetter than PAD. Stage PAW is characterized by *Phragmite australis* which is higher than stage PAD.
3. Compared with stage PAW, stage PAD has smaller *P. australis* and drier soils.
4. Stage PA + PAL is characterized by mixed dominant species of *P. australis* and *P. arundinacea* L. The plants of *P. australis* in stage PA + PAL are much smaller than stages PAW and PAD.
5. Stage PAL is characterized by the plants of *Phalaris arundinacea* L. and much drier soil. The sand content in this stage is much higher, and stage PAL is the last stage in wetland drying process.

**Figure 1:**
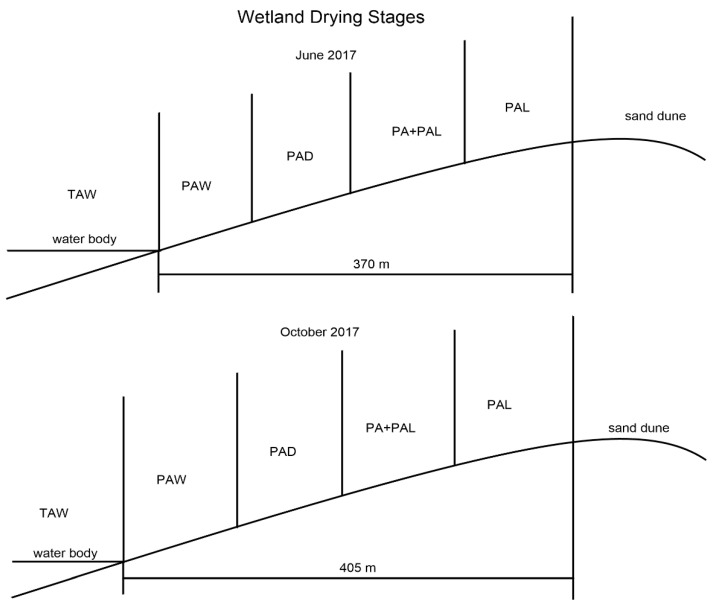
Different stages in wetland drying process. TAW, *T. augustifolia* wetland; PAW, *P. australis* wetland; PAD, *P. australis* dry land; PA + PAL, mixed grassland with *P. australis* and *P. arundinacea* L.; PAL, *P. arundinacea* L. grassland.

The water table in the lake wetland is decreasing consistently because of the increasing drought. The wetland drying process and five different successional stages are shown in Figure 1.

Considering the differences of vegetation and precipitation in June and October in Mu Us sandy land, we conducted our experiments in early June and early October [34]. In June and October 2017, vegetations in the five stages of the wetland drying process were investigated. Three plots (50 × 50 m) were selected at each stage and three subplots (10 × 10 m) were arranged in each plot. In every subplot, five quadrats were selected to conduct vegetation survey. In each quadrat, plant density, coverage, richness index, aboveground biomass and litter biomass were investigated. Vegetation data of all quadrats in each stage were taken together to calculate the vegetation data of each stage. Species diversity characteristics were calculated using the following formulas [37]:

Richness (R) = number of species in each stage(1)

(2) $$\mathrm{Shannon}\;\mathrm{Wiener}\;\mathrm{Diversity}\;\mathrm{Index}\;(H)=\sum_{1}^{N}p_{i}\times lnp_{i}$$

Plant density, coverage, diversity characteristics, aboveground biomass and litter biomass of each stage in June and October are shown in Tables S1 and S2.

### 2.2. Field Sampling and Laboratory Analysis

In June and October 2017, soil samples of each stages in wetland drying process were collected. Three plots (50 × 50 m) were selected at each stage and three subplots (10 × 10 m) were arranged in each plot. In every subplot, five quadrats were selected to conduct soil sample collection. At each quadrat, soil samples at intervals of 0–10 cm and 10–20 cm were collected by using a soil-drilling sampler with a diameter of 9 cm [38]. Soil samples at each depth were collected from each quadrat and all the samples from five quadrats in each subplot were mixed to form one sample. Nine mixed samples were collected at each depth at each stage and the final soil sample number were 90 (5 stages × 9 subplots × 2 depths). The soil samples were kept in ices bags in thermal container immediately after collection and were transported to laboratory as soon as possible. At each stage, plant root tissues and aboveground litters were collected and separated from soil samples and were then oven-dried (65 °C) and crushed in a mortar for further chemical analysis. For the soil, half of the wet soil samples were stored at 4 °C for the analysis of microbial properties. The other half of the soil samples were air dried and sieved (<2 mm) for the determination of soil physical and chemical properties.

Soil bulk density (BD) was determined by the cutting ring method [38]. Soil pH was determined by using a pH meter (HQ11d, HACH, Loveland, CO, USA) and soil electrical conductivity (EC) was determined by using a portable conductivity meter (HI993310, HANNA, Milan, Italy). A laser particle analyzer (LS-609, OMEC, Zhuhai, China) was used to determine soil particle composition (clay, silt and sand proportions). Litter and soil total nitrogen (TN) content was determined by the semi-micro Kjeldahl method. Litter and soil total phosphorus content was determined by the molybdate colorimetry method using a UV-2550 spectrophotometer (Shimadzu, Kyoto, Japan) after perchloric acid digestion. A flow analyzer (AutoAnalyzer 3, SEAL, Hamburg, Germany) was applied to determine nitrate nitrogen content and ammonium nitrogen content of soils. For the determination of soil and litter total organic carbon (TOC) content, soil and litter samples were first digested using concentrated sulfuric acid and were then determined by potassium dichromate oxidation method [39].

A chloroform fumigation-extraction method was applied for the determination of microbial biomass carbon (MBC) and nitrogen (MBN) [40,41]. Fresh soil that was equivalent to 25 g oven-dry soil was weighted and fumigated with ethanol-free CHCl_3_ (24 h, 25 °C). The soil was extracted with 100 mL 0.5 mL L^−1^ K_2_SO_4_ solution after fumigant removal. The mixture of soil and K_2_SO_4_ solution was then shaken in a reciprocal shaker (200 rpm, 60 min). At the same time of the fumigation process, a non-fumigated 25 g soil sample was also extracted with 100 mL 0.5 mL L^−1^ K_2_SO_4_ solution and shaken in the reciprocal shaker. The extracts from both fumigated and non-fumigated mixtures were filtered by Whatman No. 42 filter paper and the filtrate was stored at −15 °C and ready for further analysis. A liquid TOCII analyzer (Elementar, Hanau, Germany) was applied to determine the total organic carbon (TOC) of the extracts. The Kjeldahl method was applied for the determination of TN content of the extracts. The experimentally-derived conversion factors were 0.45 for MBC and 0.54 for MBN [42].

To determine soil microbial community structure, soil Phospholipid Fatty Acids (PLFAs) were analyzed [43]. Wet soil samples were first freeze dried, and then the 5.0 g freeze dried soils were mixed into a buffer mixture of chloroform, methanol and phosphate (1:2:0.8) for 2 h to extract lipids. The extracted lipids were then transferred to a solid-phase silica column (Agilent Technologies, Palo Alto, CA, USA) for lipids separation. By using 5 mL chloroform, 10 mL acetone and 5 mL methanol, phospholipids were separated from neutral lipids, glycolipids and polar lipids. Mild-alkaline methanolysis treatment was then conducted for the phospholipids, and the phospholipids were then dissolved in chloroform and were purified by using a solid-phase amino column (Agilent Technologies, Palo Alto, CA, USA). Fatty acid methyl esters were finally dissolved in 0.2 mL 1:1 hexane:methyl t-butyl ether (with 0.25 mg 20:0 ethyl ester mL^−1^ as an internal standard) and analyzed by using an Agilent 6890 gas chromatograph with an Agilent Ultra 2 column (Agilent Technologies). The final identification of phospholipids was then conducted according to the MIDI eukaryotic methods with Sherlock software (MIDI Inc., Newark, DE, USA). The phospholipids which were considered as indicators of bacterial groups were: i14:0, i15:0, a15:0, 16:1ω7c, i16:0, i16:1c, 17:1ω8c, 17:0cy, a17:0, i17:0, 18:1ω5c, 18:1ω7c and 19:0cy. The phospholipids which were selected as indicators of fungal group were: 16:1ω5c, 18:2ω6.9c and 18:1ω9c. All of the PLFAs including bacterial and fungal groups and other PLFAs were considered to be representative of the total PLFAs of soil microbial community [30,44].

Incubation experiment was conducted to determine soil basal microbial respiration. Fresh soil samples were first sieved (2 mm) and mixed with litter (according to the litter biomass and species in the subplot) and the soil moisture was adjusted (by regularly adding water and keeping the jars in a constant weight) to the average soil moisture of the subplot before incubation treatment [30,45]. For each treatment, 50.0 g fresh soil was added in a 500 mL glass jar and incubation lasted for 14 days at the temperature of 25 °C. At the 1st, 4th, 9th and 14th day of incubation, 15 mL headspace gas samples were collected at time intervals of 0, 30 and 60 min by using 15 mL plastic syringes. By using gas chromatography (GC 7890A, Agilent, PaloAlto, CA, USA), the gas samples were analyzed for CO_2_ concentrations. For each treatment, a linear regression was conducted between CO_2_ concentrations and time and the slope of the regression was estimated to be the instantaneous microbial respiration. The ratio of basal microbial respiration to MBC content was calculated to be microbial metabolic quotient (qCO_2_) [30,46].

### 2.3. Statistic Analysis

The differences in the soil variables with different successional stages were conducted by a one-way ANOVA with SPSS 20.0 (SPSS Corporation, Chicago, IL, USA). Pearson correlation analysis was conducted with R language 3.6.2 (Microsoft Corporation, Redmond, DC, USA).

We used the structural equation model (SEM) to analyze the direct effect of SWC on soil physicochemical properties and microbial properties. We also analyzed the indirect effect of SWC on soil microbial properties through analyses of its direct effect on soil physicochemical properties. The direct path from SWC to soil microbial properties represented the direct effect of the SWC on soil microbial properties. The direct path from SWC to soil physicochemical properties and then to the soil microbial properties represented the indirect effect of SWC on soil microbial properties through its direct effect on soil physicochemical properties.

The SEM was established by Amos 22.0 (SPSS Corporation, Chicago, IL, USA). Before analysis, all the variables were examined for a normal distribution and were log-transformed. An initial SEM model was first established according to theoretical knowledge and several parameters [minimum value of the discrepancy (CMIN), degree of freedom (DF), CMIN/DF, probability (P)] were used to determine whether initial model adequately fit the actual structure of the data. If the initial model does not fit the data, the SEM model would be corrected according to the model modification indices [47].

## 3. Results

### 3.1. Litter Biological Traits and Soil Physicochemical Properties in Wetland Drying Process

According to the results of ANOVA, different successional stages had significant effects on litter TOC, TN, TP content and litter C:N, C:P and N:P ratios (*p* < 0.01) (Table S3). From stages PAW to PAL, litter TOC content had little variation while litter TN and TP content decreased consistently in June and October (Tables S4 and S5). From stages PAW to PAL, litter C:N ratio and C:P ratio increased consistently in June and October. Litter TOC, TN and TP contents in TAW were significantly lower than stages from PAW to PAL (*p* < 0.05) (Tables S4 and S5).

Results of ANOVA showed that different successional stages had significant effects on SWC, BD, soil clay, silt and sand content (*p* < 0.01) (Table S6). From stages TAW to PAL, SWC, soil clay contents and soil silt contents decreased consistently, while soil bulk density and soil sand contents increased in June and October. Soil pH had few variations in wetland drying process in June and October (Figures S1 and S2). The sub soils (10–20 cm) had nearly the same trends in physical properties compared with the top soils (0–10 cm). However, SWC and soil contents were lower while BD and soil sand contents were higher in sub soils (10–20 cm) as compared with top soils (0–10 cm) (Figures S1 and S2).

Results of ANOVA showed that different successional stages had significant effects on SOC, TN, ammonium nitrogen, nitrate nitrogen and TP content and soil C:N, C:P and N:P ratios (*p* < 0.01) (Table S6). From stages PAW to PAL, SOC contents, soil TN contents, soil ammonium nitrogen contents, soil C:N ratios and soil C:P ratios decreased consistently, while soil nitrate nitrogen contents increased consistently in June and October. Soil TP contents were much higher in TAW than in other stages (*p* < 0.05) and had little variation from stages PAW to PAL (Figures S3 and S4).

### 3.2. Microbial Properties in Different Stages of Wetland Drying Process

**Figure 2:**
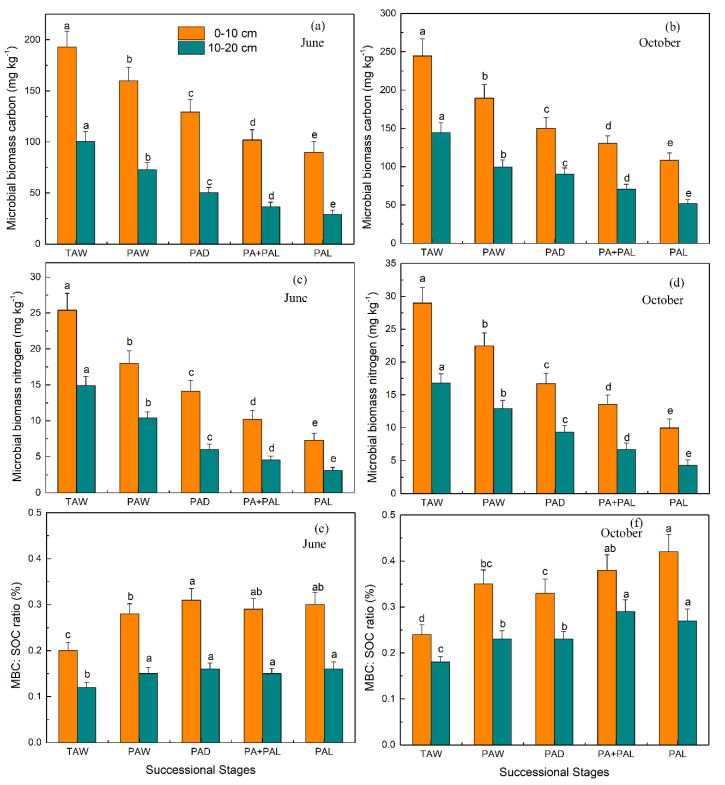
Microbial biomass and microbial quotient (MBC:SOC ratio) in the wetland drying process in June (**a**,**c**,**e**) and October (**b**,**d**,**f**). Values are Mean ± SE (*n* = 9). Different letters over the bars indicate statistically significant differences between different stages. TAW, *T. augustifolia* wetland; PAW, *P. australis* wetland; PAD, *P. australis* dry land; PA + PAL, mixed grassland with *P. australis* and *P. arundinacea* L.; PAL, *P. arundinacea* L. grassland.

The results of the ANOVA showed that different successional stages had significant effects on MBC, MBN, MBC:SOC ratio, basal respiration, microbial metabolic quotient, bacteria abundance, fungi abundance and fungi:bacteria ratio (*p* < 0.01) (Table S7). Soil MBC and MBN contents decreased consistently, while MBC:SOC ratios increased in the wetland drying process (Figure 2). From stages TAW to PAL, in the top soil (0–10 cm), soil MBC content decreased from 192.9 mg kg^−1^ to 90.0 mg kg^−1^ in June and decreased from 244.5 mg kg^−1^ to 108.5 mg kg^−1^ in October; soil MBN content from 25.4 mg kg^−1^ to 7.30 mg kg^−1^ in June and decreased from 29.0 mg kg^−1^ to 9.95 mg kg^−1^ in October; From stages TAW to PAL, in the top soil (0–10 cm), soil MBC:SOC ratio increased from 0.20% to 0.30% in June and increased from 0.24% to 0.42% in October (Figure 2). From stages PAW to PAL, soil MBC:SOC ratios had little variation in June (0.28–0.30%) and increased a little in October (0.33–0.37%) (Figure 2).

**Figure 3:**
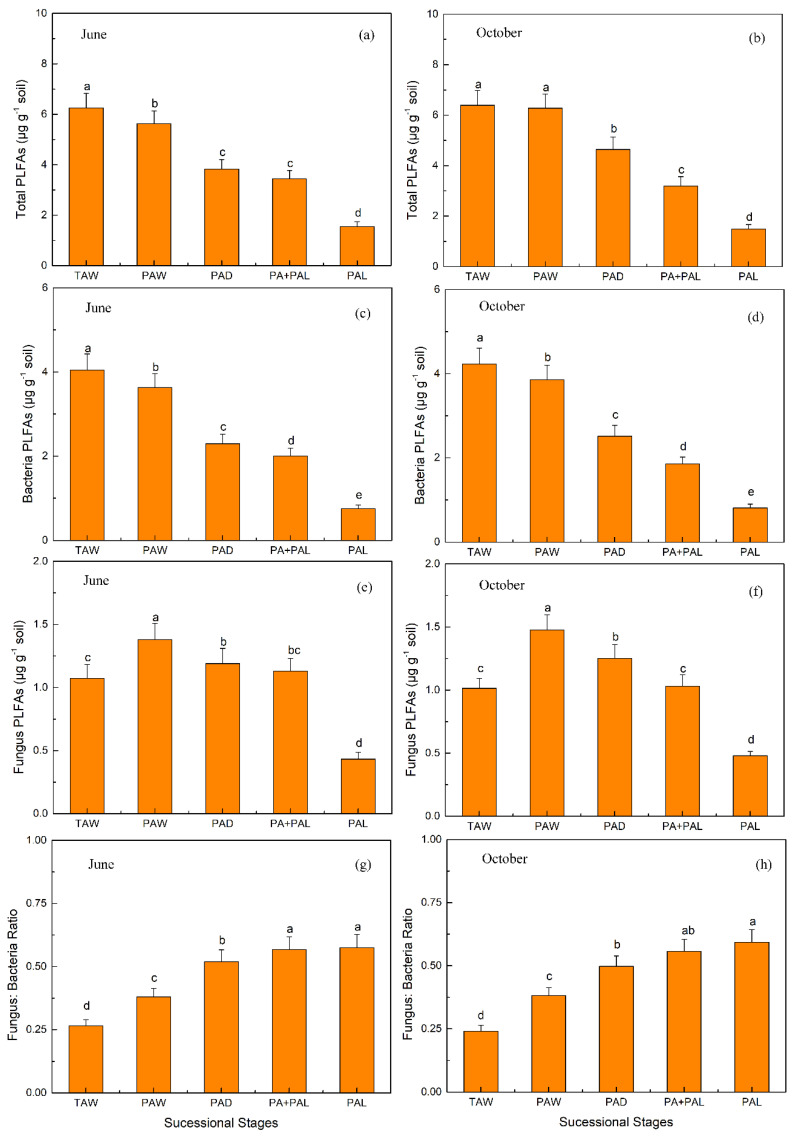
Microbial community structure of top soil (0–10 cm) derived from phospholipid fatty acid (PLFAs) analysis at different stages of wetland drying process in June (**a**,**c**,**e**,**g**) and October (**b**,**d**,**f**,**h**). Values are Mean ± SE (*n* = 9). Different letters over the bars indicate statistically significant differences between different stages. TAW, *T. augustifolia* wetland; PAW, *P. australis* wetland; PAD, *P. australis* dry land; PA + PAL, mixed grassland with *P. australis* and *P. arundinacea* L.; PAL, *P. arundinacea* L. grassland.

Total, bacterial and fungal PLFA decreased and fungi:bacteria ratio increased in the wetland drying process in June and October (Figure 3). Bacterial PLFA decreased from 4.05 to 0.75 μg g^−1^ in June and decreased from 4.23 to 0.81 μg g^−1^ in October; fungal PLFA decreased from 1.07 to 0.43 μg g^−1^ in June and decreased from 1.01 to 0.48 μg g^−1^ in October; fungi:bacteria ratio increased from 0.27 to 0.57 in June and increased from 0.24 to 0.56 in October (Figure 3).

**Figure 4:**
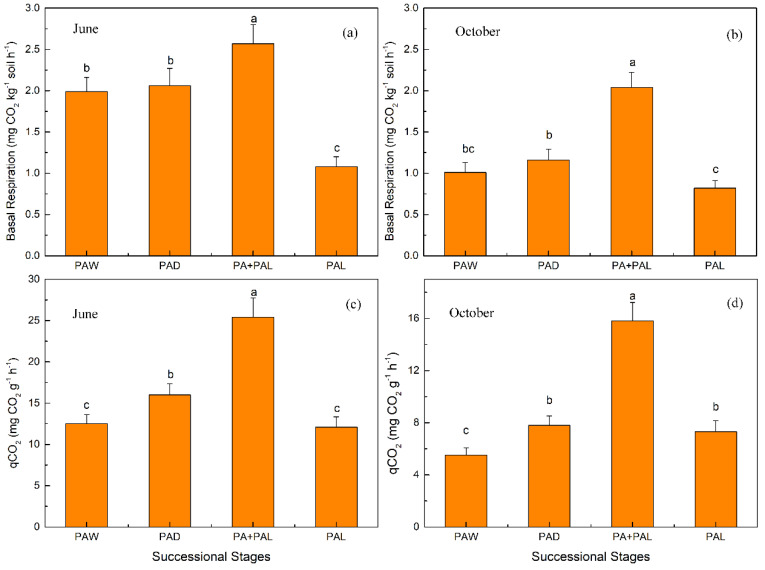
Soil basal respiration and microbial metabolic quotient (qCO_2_) under different successional stages in June (**a**,**c**) and October (**b**,**d**). Values are Mean ± SE (*n* = 9). Different letters over the bars indicate statistically significant differences between different stages. TAW, *T. augustifolia* wetland; PAW, *P. australis* wetland; PAD, *P. australis* dry land; PA + PAL, mixed grassland with *P. australis* and *P. arundinacea* L.; PAL, *P. arundinacea* L. grassland.

Soil basal respiration and microbial metabolic quotient (qCO_2_) of each stage in June and October are shown in Figure 4. Soil basal respiration ranged from 1.08 to 2.57 mg CO_2_ kg^−1^ soil h^−1^ in June and range from 0.82 to 2.04 mg CO_2_ kg^−1^ soil h^−1^ in October. Soil qCO_2_ ranged from 12.1 to 25.4 mg CO_2_ g^−1^ h^−1^ in June and ranged from 5.5 to 15.8 mg CO_2_ g^−1^ h^−1^ in October. Soil basal respirations and qCO_2_ showed a trend of first increasing and then decreasing from PAW to PAL in June and October. Soil basal respiration and qCO_2_ both increased from stages PAW to PA + PAL and then decreased when transferred to PAL stage. Soils in PA + PAL stage had the highest values of soil basal respiration and qCO_2_ in June and October (Figure 4).

### 3.3. Correlation between Soil Physicochemical Properties and Soil Microbial Properties

**Figure 5:**
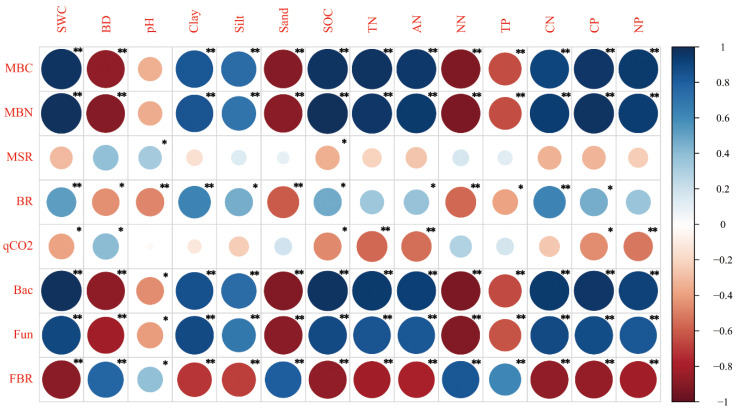
Correlation analysis between microbial properties and soil physical chemical properties in June. SWC, soil water content; BD, soil bulk density; Clay, soil clay content; Silt, soil silt content; Sand, soil sand content; SOC, soil organic carbon; TN, total nitrogen content; AN, ammonium nitrogen content; NN, nitrate nitrogen content; TP, soil total phosphorus content; CN, soil C:N ratio; CP, soil C:P ratio; NP, soil N:P ratio; MBC, microbial biomass carbon; MBN, microbial biomass nitrogen; MSR, MBC:SOC ratio; BR, basal respiration; qCO2, microbial metabolic quotient; Bac, bacteria abundance; fun, fungi abundance; FBR, fungi:bacteria ratio. * *p* < 0.05, ** *p* < 0.01.

**Figure 6:**
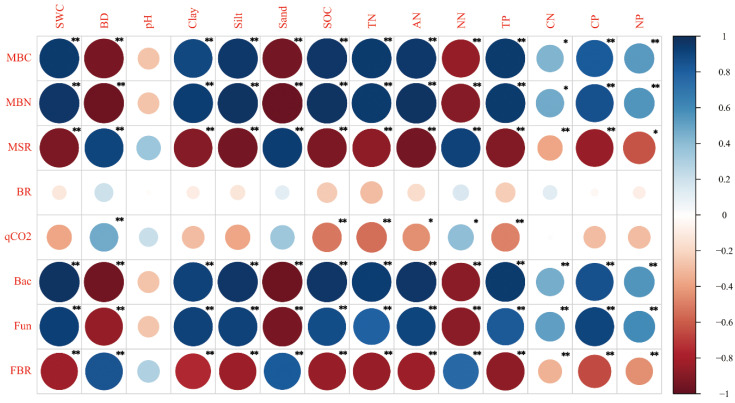
Correlation analysis between microbial properties and soil physical chemical properties in October. SWC, soil water content; BD, soil bulk density; Clay, soil clay content; Silt, soil silt content; Sand, soil sand content; SOC, soil organic carbon; TN, total nitrogen content; AN, ammonium nitrogen content; NN, nitrate nitrogen content; TP, soil total phosphorus content; CN, soil C:N ratio; CP, soil C:P ratio; NP, soil N:P ratio; MBC, microbial biomass carbon; MBN, microbial biomass nitrogen; MSR, MBC:SOC ratio; BR, basal respiration; qCO2, microbial metabolic quotient; Bac, bacteria abundance; fun, fungi abundance; FBR, fungi:bacteria ratio. * *p* < 0.05, ** *p* < 0.01.

The correlation results between soil microbial properties and physicochemical properties in June are presented in Figure 5 and Figure 6. The levels of MBC, MBN, bacteria abundance and fungi abundance showed a positive correlation with the levels of SWC, clay content, silt content, SOC, TN, ammonium nitrogen and soil C:N, C:P and N:P ratios, and showed a negative correlation with levels of BD, sand content and nitrate nitrogen in June and October (*p* < 0.01) (Figure 5 and Figure 6). In contrast, the levels of fungi:bacteria ratios showed a negative correlation with the levels of SWC, clay content, silt content, SOC, TN, ammonium nitrogen and soil C:N, C:P and N:P ratios, and showed a positive correlation with levels of BD, sand content and nitrate nitrogen in June and October (*p* < 0.01) (Figure 5 and Figure 6).

The levels of MBC:SOC ratios showed a positive correlation with levels of BD, sand and nitrate nitrogen and showed a negative correlation with levels of SWC, clay, silt, SOC, TN, ammonium nitrogen, C:N ratios, C:P ratios and N:P ratios in October (*p* < 0.01) (Figure 6). Soil basal respiration had significant positive correlations with SWC, soil clay content and soil C:N ratio, and had significant negative correlations with pH, soil sand and nitrate nitrogen content in June (*p* < 0.01) (Figure 5). In contrast, soil basal respiration had weak correlation with soil physicochemical properties in October (Figure 6). Microbial metabolic quotient (qCO_2_) had positive correlations with soil BD in June and October (*p* < 0.05). Moreover, qCO_2_ had negative correlations with SOC, soil TN and ammonium nitrogen contents in June and October (*p* < 0.05) (Figure 5 and Figure 6).

### 3.4. Direct and Indirect Effects of SWC on Microbial Properties by Influencing Soil Properties

**Figure 7:**
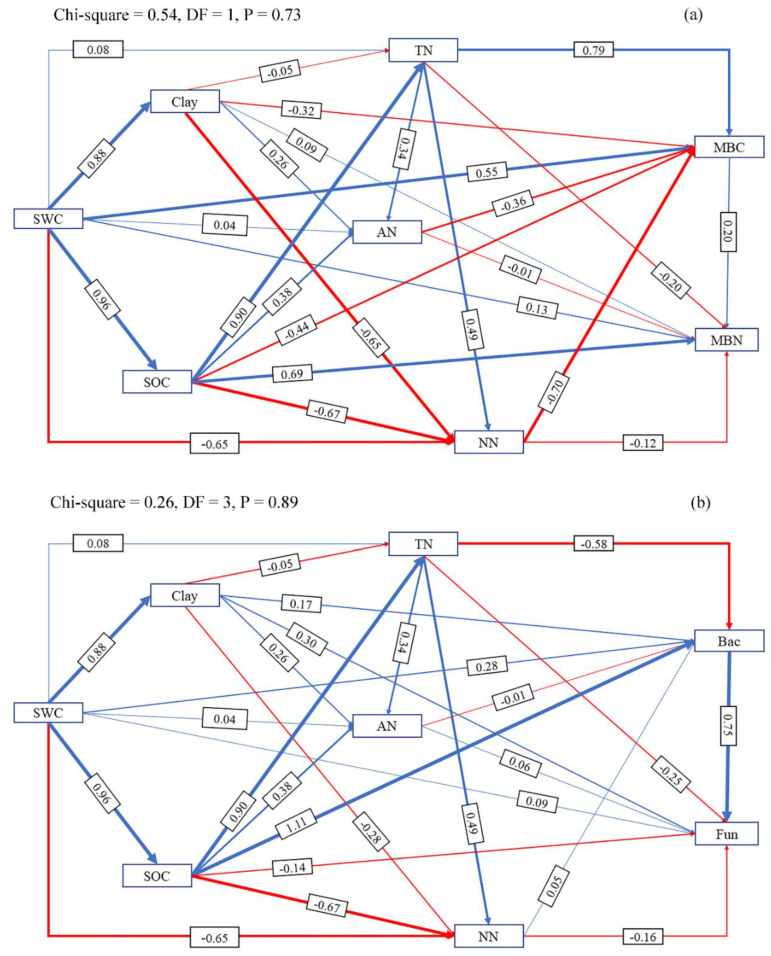
Direct and indirect effects of soil water content (SWC) on microbial biomass carbon (MBC) and microbial biomass nitrogen (MBN) by influencing soil physical and chemical properties were shown in (**a**). And the direct and indirect effects of soil water content (SWC) on bacteria abundance and fungi abundance by influencing soil physical and chemical properties were shown in (**b**). Blue lines indicate positive direct effects and red lines indicate negative direct effects. SWC, soil water content; Clay, soil clay content; SOC, soil organic carbon; TN, soil total nitrogen content; NN, soil nitrate nitrogen; AN, soil ammonium nitrogen content; MBC, microbial biomass carbon; MBN, microbial biomass nitrogen; Bac, bacteria abundance; Fun, fungi abundance.

**Figure 8:**
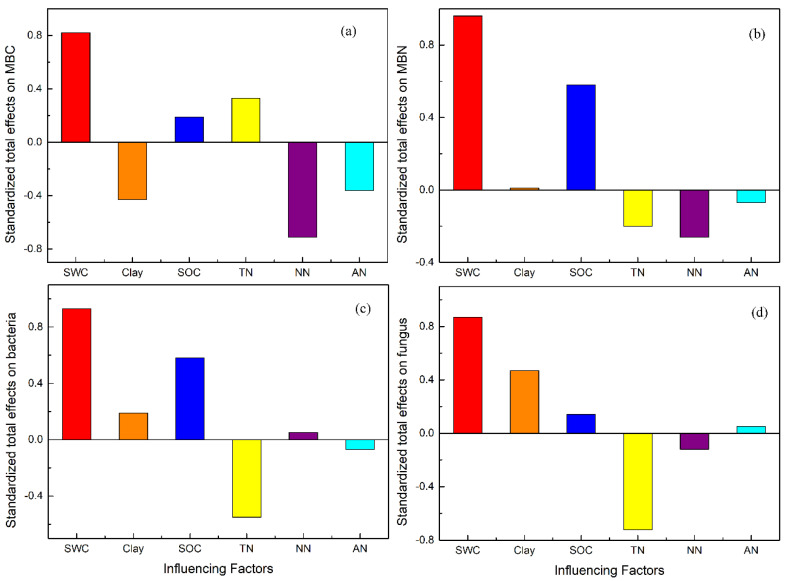
Standardized total effects on microbial biomass carbon (MBC) (**a**), microbial biomass nitrogen (MBN) (**b**), bacteria abundance (**c**) and fungi abundance (**d**). SWC, soil water content; Clay, soil clay content; SOC, soil organic carbon; TN, soil total nitrogen content; NN, soil nitrate nitrogen; AN, soil ammonium nitrogen content.

According to the results of SEM, paths from SWC to soil physicochemical properties and then to soil MBC and MBN contents are shown in Figure 7. The results showed that soil TN content and SWC had significant positive direct effects on MBC content while SOC content had significant positive direct effects on MBN (Figure 7). For the standardized total effects, MBC was mainly affected by SWC (0.82) and nitrate nitrogen (−0.70), while MBN was mainly affected by SWC (0.96) and SOC content (0.58) (Figure 8).

According to the results of SEM, paths from SWC to soil physicochemical properties and then to soil bacteria and fungi abundance are shown in Figure 7. The results showed that SOC content and SWC had significant positive direct effects on bacteria abundance, while soil TN content had significant negative effects on bacteria abundance. Soil fungi abundance was less affected by environmental changes compared with bacteria and mainly received positive direct effects from soil clay content and negative direct effects from soil TN content (Figure 7). For the standardized total effects, bacteria abundance was mainly affected by SWC (0.93), SOC content (0.58) and soil TN content (−0.55) and fungi abundance was mainly affected by SWC (0.87), soil TN content (−0.72) and soil clay content (0.47) (Figure 8).

## 4. Discussion

In this study, contents of soil MBC and MBN decreased in the declining process of wetland soil in the Mu Us desert (Figure 2). It was shown that soil MBC content had a significant positive correlation with SWC, SOC and litter TOC contents (*p* < 0.05) (Figure 5, Figure 6 and Figure S5) and soil MBN content had a significant positive correlation with SWC and SOC (*p* < 0.01) (Figure 5 and Figure 6). Results of SEM also showed the predominant effects of SWC on soil MBC and MBN contents (Figure 8). The decrease of soil MBC and MBN contents was attributed to the decrease of SWC, SOC content and litter input. Water is critical for the living of microbes and soil water content had significant effects on microbes in both dry and wet environment [5,48]. On the one hand, although soil aeration conditions were ameliorated under lower SWC, reduced water availability could limit substrate diffusivity and accessibility for soil microbes, and thus inhibited microbial growth [49,50]. On the other hand, under lower water potentials, water-stress response of soil microbes will be triggered, in the form of osmotic regulation or drought avoidance through dormancy [10]. In this study, the decrease of SOC and litter input could also lead to the decrease of soil microbial biomass, since SOC and litter were the substrates necessary for the living of microorganism [12,30].

Soil microbial quotients (MBC:SOC) were much lower in virgin wetland stage (TAW) and higher in dryland stages (PAW to PAL) (Figure 2). In this study, MBC:SOC ratios of top soils (0–10 cm) were in a range of 0.20–0.30% in June and in a range of 0.24–0.42% in October (Figure 2). It was reported that the soil MBC:SOC ratios of woodland, shrubland and cropland were in a range of 6.0–10.0% in the Danjiangkou Reservoir [30]. Anderson et al. (2010) reported that the microbial quotient was 2.3% for monoculture soils and 2.9% for crop rotation soils [13]. Compared with studies of Deng et al. (2016) and Anderson et al. (2010), it can be shown that MBC:SOC ratios in Mu Us sandy land were much lower [13,30]. Lower MBC:SOC ratios indicated that soil microbes were stressed because of lower SOC and MBC contents and higher sand content in soil [26]. Although soil MBC and MBN contents decreased, the MBC:SOC ratios did not decrease in wetland drying process and the MBC:SOC ratios was higher in PA + PAL and PAL than in TAW and PAW (Figure 2). The maintenance of MBC:SOC ratios were attributed to the following reasons: (1) soil C:N ratio decreased in wetland drying process and higher substrate quality (lower soil C:N ratio) could promote microbial assimilation of carbon and thus lead to higher MBC:SOC ratios [51]; (2) microbes could adapt to drier environments by synthesizing solutes such as polyols and amino acids [52] and by community composition shifting (higher fungi:bacteria ratio) [50]; (3) soils had higher fungi:bacteria ratios under drier conditions and fungi were reported to have stronger ability to conserve SOC (Six et al., 2006).

According to the result, both fungi and bacteria abundance decreased and fungi:bacteria increased in the wetland drying process (Figure 3). The decreases of fungi and bacteria abundance were attributed to the decrease of SWC and substrate (SOC and litter biomass). Fungi had a smaller decreasing scale compared with bacteria in response to the drying of wetland, leading to an increased fungi:bacteria ratio. The results suggest that soils with increasing aridity favor a fungal-rich microbial community, since fungi were able to overcome better the disadvantages of drier conditions than bacteria [22,53,54], with hyphae that may cross air-filled soil pores to access nutrients and water [55]. Previous studies showed that the relative abundance of *Acidobacteria* declined linearly as aridity increased while the relative abundance of major fungal phyla did not change with aridity [6,55].

In this study, soil basal respiration showed a trend of first increasing and then decreasing in wetland drying process (Figure 4). The changes of soil aeration conditions and quantity and quality of substrates were the main factors influencing soil respiration [56,57]. Soil in virgin wetland was under anaerobic condition and the lack of oxygen limited the respiration of microbes [58,59]. When water declined, soils shifted from an anaerobic condition to an aerobic condition and basal respiration of soil microbes increased rapidly [7,9]. In the wetland drying process, vegetation biomass and litter input was also a major factor affecting soil respiration [60]. In the last stage of wetland drying process, the decrease of vegetation biomass and litter input led to the decrease of substrates for soil respiration and then led to the decrease of soil respiration [29].

Soil qCO_2_ showed a trend of first increasing and then decreasing in soil drying process and the highest value was achieved in PA + PAL (Figure 4). Soil tended to have low qCO_2_ values under suitable environment conditions (moderate water content, less sand contents and less hazardous materials such as heavy metal or organic pollutants) and with sufficient nutrient supply [24,27]. In this study, qCO_2_ was positively correlated with soil sand content and negatively correlated with soil clay content (Figure 6), since finer soil texture protects soil microbial biomass against degradation and limits organic matter mineralization, and thus had lower qCO_2_ values [61]. Substrate C:N and C:P ratio and soil community structure can also influence soil qCO_2_. Higher soil and litter C:N and C:P usually indicate lower soil nutrient supply and low efficiency of microbial biomass carbon utilization, which lead to higher qCO_2_ [24,28] and fungi had higher carbon utilization efficiency and lower qCO_2_ [11,31]. Previous study showed that lower soil and litter C:N ratio, higher litter N concentration and higher fungi:bacteria ratio could lead to lower qCO_2_ [12,24,30]. In this study, qCO_2_ was positively correlated with litter C:N ratio and negatively correlated with litter N concentration (Figure S6). With increasing litter C:N ratio, microbial carbon use efficiency decreases because the microorganisms do not have enough N to build up as much biomass as the C concentration would allow them [62,63,64]. Although previous studies showed that qCO_2_ was positively correlated with soil C:N ratio and was positively correlated with fungi:bacteria ratios [12,30], qCO_2_ had a weak correlation with the soil C:N ratio and was positively correlated with fungi:bacteria ratio in this study (Figures S5 and S6). For the relationship between qCO_2_ and soil microbial structure, previous studies showed that a higher fungi:bacteria ratio could lead to lower qCO_2_ values [30], because fungi are known to have slower biomass turnover rates and lower qCO_2_ values [11,65]. However, in this study, qCO_2_ had quite weak correlations with fungi:bacteria ratios (Figure S7) and the effects of the microbial community structure on qCO_2_ was not clear. The increase of qCO_2_ was more attributed to the decrease of clay content and litter N content and the increase of litter C:N ratio, regardless of the decrease of soil C:N ratio and bacteria:fungi ratio.

A shift in microbial biomass and community structure after wetland drying may have significant impacts on soil C cycles [45,66]. In this study, fungi:bacteria ratios were positively correlated with soil basal respiration and qCO_2_ (Figure S7). However, according to previous study, fungi are known to have slower biomass turnover rates and lower qCO_2_ than bacteria [11,65]. This indicated that changes of soil basal respiration and qCO_2_ in this study were mainly attributed to changes of environmental conditions (soil aeration condition, SWC, clay content), rather than by community structure. Moreover, this study also showed negative correlations between MBC:SOC ratios and soil basal respiration (Figure S7), indicating that soil MBC:SOC ratio was closely related with microbial activity [67]. Soil microbial metabolic quotient (qCO_2_) was positively correlated with fungi:bacteria ratio in this study (Figure S7). However, according to a previous study, MBC:SOC ratio is negatively correlated with qCO_2_, since the decreased available substrate (MBC:SOC ratio) could lead to lower microbial substrate use efficiency and higher qCO_2_ [13,26]. Based on the results of this study, the relationship between MBC:SOC ratios and qCO_2_ needs further research.

## 5. Conclusions

The drying of wetland led to a decrease of soil MBC content, MBN content and fungi and bacterial abundance, and an increase of fungi:bacteria ratios. The decrease of MBC and MBN contents were attributed to the decline of substrate (SOC, litter) and SWC, and the increase of fungi:bacteria ratios was attributed to the adaptation of microbes to the drier environment. Wetland drying also led to increased soil basal respiration, which was attributed to the amelioration of soil aeration condition and increased qCO_2_ which was attributed to lower soil clay content and litter N concentration. The MBC:SOC ratios were higher under drier soil conditions than under virgin wetland, which were attributed to the stronger C conserve ability of fungi than bacteria. The wetland drying process exacerbated soil C loss by strengthening the heterotrophic respiration; however, the exact effects of soil microbial community structure on microbial C mineralization were not clear in this study and need further research.

## Supplementary Materials

The following are available online at https://www.mdpi.com/1660-4601/17/12/4199/s1, Figure S1: Soil water content (a), bulk density (b), pH (c), soil clay content (d), soil silt content (e) and soil sand content (f) in different stages of wetland drying process in June, Figure S2: Soil water content (a), bulk density (b), pH (c), soil clay content (d), soil silt content (e) and soil sand content (f) in different stages of wetland drying process in October, Figure S3: Soil organic carbon (a), total nitrogen content (b), ammonium nitrogen (c), nitrate nitrogen (d), total phosphorus content (e), soil C: N ratio (f), soil C: P ratio (g) and soil N: P ratio (h) in different stages of wetland drying process in June, Figure S4: Soil organic carbon (a), total nitrogen content (b), ammonium nitrogen (c), nitrate nitrogen (d), total phosphorus content (e), soil C: N ratio (f), soil C: P ratio (g) and soil N: P ratio (h) in different stages of wetland drying process in October, Figure S5: The relationships between soil MBC (a) and MBN (b) contents and litter total organic carbon content, Figure S6: The relationships between soil microbial metabolic quotient (qCO_2_) with soil C: N ratio (a), litter total nitrogen content (b) and litter C: N ratio (c), Figure S7: The relationships between soil basal respiration and microbial quotient (MBC: SOC ratio) (a) and fungi: bacteria ratio (c) and the relationships between soil microbial metabolic quotient (qCO_2_) (b) and microbial quotient (MBC: SOC ratio) and fungi: bacteria ratio (d),Table S1: Characteristics of vegetation during different successional stages in June, Table S2: Characteristics of vegetation during different successional stages in October, Table S3: Summary statistics (F statistic and probability level) of a one-way ANOVA on the effects of different succession stages on litter chemical properties in June and October, Table S4: Chemical properties of litters in different stages of wetland drying process in June, Table S5: Chemical properties of litters in different stages of wetland drying process in October, Table S6: Summary statistics (F statistic and probability level) of a one-way ANOVA on the effects of different succession stages on soil physical and chemical properties in June and October, Table S7: Summary statistics (F statistic and probability level) of a one-way ANOVA on the effects of different succession stages soil microbial properties in June and October.
