# Supplementary material for: Soil Microbial Community and Its Interaction with Soil Carbon Dynamics Following a Wetland Drying Process in Mu Us Sandy Land
Source: Int J Environ Res Public Health. 2020 Jun 12;17(12):4199. doi: 10.3390/ijerph17124199 (PMC7345046; doi:10.3390/ijerph17124199)
Supplement: Supplementary file 1 [file ijerph-17-04199-s001.pdf]

Table S1. Characteristics of vegetation during different successional stages in June.

Stages	Density (m^{-2})	Coverage (%)	R	H	Biomass (g m^{-2})	Litter (g m^{-2})
TAW	60.1 ± 7.5	85.6 ± 7.2	7	1.05	1252.3 ± 81.3	211.5 ± 18.5
PAW	70.3 ± 11.4	75.2 ± 6.5	8	1.16	954.6 ± 71.2	71.2 ± 12.3
PAD	53.1 ± 7.1	73.2 ± 5.5	8	1.22	824.5 ± 51.3	68.5 ± 9.1
PA + PAL	48.5 ± 6.2	71.2 ± 4.9	9	1.36	645.1 ± 44.4	54.1 ± 8.5
PAL	40.3 ± 5.1	62.4 ± 5.3	5	0.81	491.6 ± 42.6	40.5 ± 6.8

Note: TOC, total organic carbon; TN, total nitrogen; TP, total phosphorus; C: N, TOC: TN ratio; C: P, TOC: TP ratio; N: P ratio, TN: TP ratio; H, Shannon-Wiener Diversity Index; R, richness index. Values were means ± SE (n = 9). TAW, T. *augustifolia* wetland; PAW, P. *australis* wetland; PAD, P. *australis* dry land; PA+PAL, mixed grassland with P. *australis* and P. *arundinacea* L.; PAL, P. *arundinacea* L. grassland.

Table S2. Characteristics of vegetation during different successional stages in October.

Stages	Density	Coverage	R	H	Biomass (g m^{-2})	Litter (g m^{-2})
TAW	232.6 ± 21.1	87.9 ± 8.6	6	1.01	1549.3 ± 175.2	265.6 ± 23.4
PAW	298.6 ± 24.3	91.2 ± 8.9	6	1.08	1120.4 ± 122.6	103.1 ± 11.5
PAD	154.7 ± 18.5	85.4 ± 5.5	7	1.18	910.5 ± 95.6	85.6 ± 9.8
PA+PAL	205.6 ± 24.1	82.6 ± 5.1	10	1.31	874.6 ± 84.9	81.1 ± 8.7
PAL	132.5 ± 19.4	78.6 ± 4.4	5	0.78	866.8 ± 75.4	77.4 ± 8.2

Note: TOC, total organic carbon; TN, total nitrogen; TP, total phosphorus; C: N, TOC: TN ratio; C: P, TOC: TP ratio; N: P ratio, TN: TP ratio; H, Shannon-Wiener Diversity Index; R, richness index. Values are Means ± SE (n = 9). TAW, T. *augustifolia* wetland; PAW, P. *australis* wetland; PAD, P. *australis* dry land; PA+PAL, mixed grassland with P. *australis* and P. *arundinacea* L.; PAL, P. *arundinacea* L. grassland.

Table S3. Summary statistics (F statistic and probability level) of a one-way ANOVA on the effects of different succession stages on litter chemical properties in June and October.

	June		October	
	F	p	F	p
Litter TOC	24.4	<0.01	22.9	<0.01
Litter TN	27.4	<0.01	69.2	<0.01
Litter TP	57.1	<0.01	68.1	<0.01
Litter C: N	16.8	<0.01	51.8	<0.01
Litter C: P	23.4	<0.01	11.5	<0.01
Litter N: P	1.98	0.12	15.6	<0.01

Note: TOC, total organic carbon; TN, total nitrogen; TP, total phosphorus; C: N, TOC: TN ratio; C: P, TOC: TP ratio; N: P ratio, TN: TP ratio.

Table S4. Chemical properties of litters in different stages of wetland drying process in June.

	TOC	TN	TP	C: N	C: P	N: P
TAW	276.1 ± 22.5 ^{b}	9.81 ± 0.91 ^{b}	1.05 ± 0.091 ^{b,c}	28.1 ± 2.52 ^{d}	264.0 ± 22.4 ^{c}	9.38 ± 0.85 ^{a}
PAW	361.6 ± 30.2 ^{a}	12.2 ± 1.11 ^{a}	1.28 ± 0.013 ^{a}	29.7 ± 2.71 ^{c,d}	282.9 ± 25.6 ^{c}	9.57 ± 0.91 ^{a}
PAD	347.7 ± 29.8 ^{a}	10.1 ± 0.95 ^{b}	1.07 ± 0.095 ^{b}	34.7 ± 3.12 ^{b,c}	320.4 ± 28.5 ^{b}	9.35 ± 0.88 ^{a}
PA+PAL	362.3 ± 32.3 ^{a}	9.20 ± 0.88 ^{b,c}	1.04 ± 0.096 ^{b,c}	39.6 ± 3.33 ^{a,b}	348.7 ± 30.5 ^{a,b}	8.86 ± 0.82 ^{a}
PAL	357.7 ± 31.3 ^{a}	8.70 ± 0.83 ^{c}	0.98 ± 0.087 ^{c}	41.1 ± 3.55 ^{a}	360.2 ± 32.6 ^{a}	8.77 ± 0.78 ^{a}

Note: TOC, total organic carbon; TN, total nitrogen; TP, total phosphorus; C: N, TOC: TN ratio; C: P, TOC: TP ratio; N: P ratio, TN: TP ratio. Values are Mean ± SE (n = 9). Different letters indicate statistically significant differences between different stages. TAW, T. *augustifolia* wetland; PAW, P. *australis* wetland; PAD, P. *australis* dry land; PA+PAL, mixed grassland with P. *australis* and P. *arundinacea* L.; PAL, P. *arundinacea* L. grassland.

Table S5. Chemical properties of litters in different stages of wetland drying process in October.

	TOC	TN	TP	C: N	C: P	N: P
TAW	296.6 ± 25.6 [b]	9.26 ± 0.87 [b]	0.97 ± 0.088 [b,c]	32.9 ± 2.85 [c]	308.5 ± 28.5 [c]	9.37 ± 0.91 [b,c]
PAW	375.3 ± 32.3 [a]	13.1 ± 1.12 [a]	1.20 ± 0.101 [a]	28.8 ± 2.41 [d]	314.0 ± 28.4 [c]	10.9 ± 0.99 [a]
PAD	360.9 ± 29.5 [a]	10.9 ± 0.91 [b]	1.13 ± 0.095 [b,c]	33.0 ± 2.93 [c]	321.9 ± 29.6 [b,c]	9.77 ± 0.95 [b]
PA+PAL	371.0 ± 33.2 [a]	9.72 ± 0.87 [b,c]	1.08 ± 0.099 [b]	38.1 ± 3.42 [b]	342.3 ± 31.2 [a,b]	9.00 ± 0.85 [c]
PAL	366.6 ± 30.5 [a]	9.11 ± 0.83 [c]	1.00 ± 0.092 [c]	40.5 ± 3.62 [a]	364.6 ± 32.4 [a]	9.00 ± 0.81 [c]

Note: TOC, total organic carbon; TN, total nitrogen; TP, total phosphorus; C: N, TOC: TN ratio; C: P, TOC: TP ratio; N: P ratio, TN: TP ratio. Values are Mean ± SE (n = 9). Different letters indicate statistically significant differences between different stages. TAW, T. *augustifolia* wetland; PAW, P. *australis* wetland; PAD, P. *australis* dry land; PA+PAL, mixed grassland with P. *australis* and P. *arundinacea* L.; PAL, P. *arundinacea* L. grassland.

Table S6. Summary statistics (F statistic and probability level) of a one-way ANOVA on the effects of different succession stages on soil physical and chemical properties in June and October.

	June		October	
	F	*p*	F	*p*
SWC	369.4	<0.01	88.1	<0.01
BD	64.8	<0.01	79.9	<0.01
pH	3.74	0.02	3.12	0.03
Clay	32.8	<0.01	10.5	<0.01
Silt	18.7	<0.01	22.3	<0.01
Sand	49.9	<0.01	12.1	<0.01
SOC	221.9	<0.01	230.7	<0.01
TN	259.4	<0.01	135.8	<0.01
TP	139.2	<0.01	29.6	<0.01
AN	201.3	<0.01	89.1	<0.01
NN	128.9	<0.01	37.4	<0.01
C: N	42.7	<0.01	76.5	<0.01
C: P	92.8	<0.01	44.5	<0.01
N: P	48.7	<0.01	6.05	<0.01

Note: SWC, soil water content; BD, soil bulk density; Clay, soil clay content; Silt, soil silt content; Sand, soil sand content; SOC, soil organic carbon; TN, total nitrogen content; AN, ammonium nitrogen content; NN, nitrate nitrogen content; TP, soil total phosphorus content; CN, soil C: N ratio; CP, soil C: P ratio; NP, soil N: P ratio.

Table S7. Summary statistics (F statistic and probability level) of a one-way ANOVA on the effects of different succession stages soil microbial properties in June and October.

	June		October	
	F	*p*	F	*p*
MBC	306.9	<0.01	573.2	<0.01
MBN	524.1	<0.01	304.6	<0.01
MBC: SOC	35.2	<0.01	25.5	<0.01
BR	131.6	<0.01	53.2	<0.01
qCO_2	80.5	<0.01	80.5	<0.01
Bac	485.8	<0.01	419.2	<0.01
Fun	132.6	<0.01	113.1	<0.01
FBR	195.7	<0.01	65.4	<0.01

Note: MBC, microbial biomass carbon; MBN, microbial biomass nitrogen; BR, basal respiration; qCO2, microbial metabolic quotient; Bac, bacteria abundance; fun, fungi abundance; FBR, fungi: bacteria ratio.

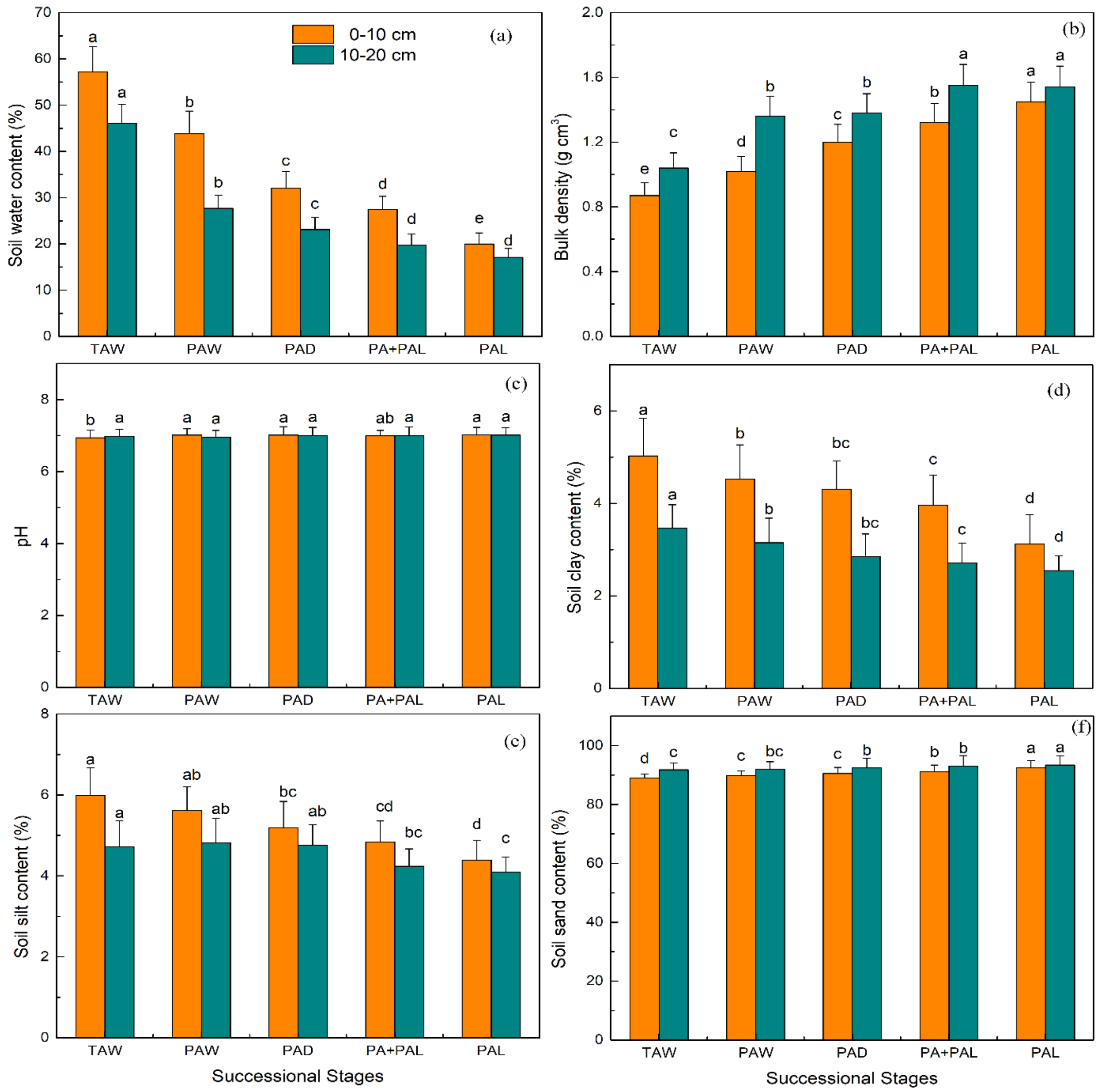

Figure S1. Soil water content (**a**), bulk density (**b**), pH (**c**), soil clay content (**d**), soil silt content (**e**) and soil sand content (**f**) in different stages of wetland drying process in June. Values are Mean ± SE (*n* = 9). Different letters over the bars indicate statistically significant differences between different stages. TAW, T. *augustifolia* wetland; PAW, P. *australis* wetland; PAD, P. *australis* dry land; PA+PAL, mixed grassland with P. *australis* and P. *arundinacea* L.; PAL, P. *arundinacea* L. grassland.

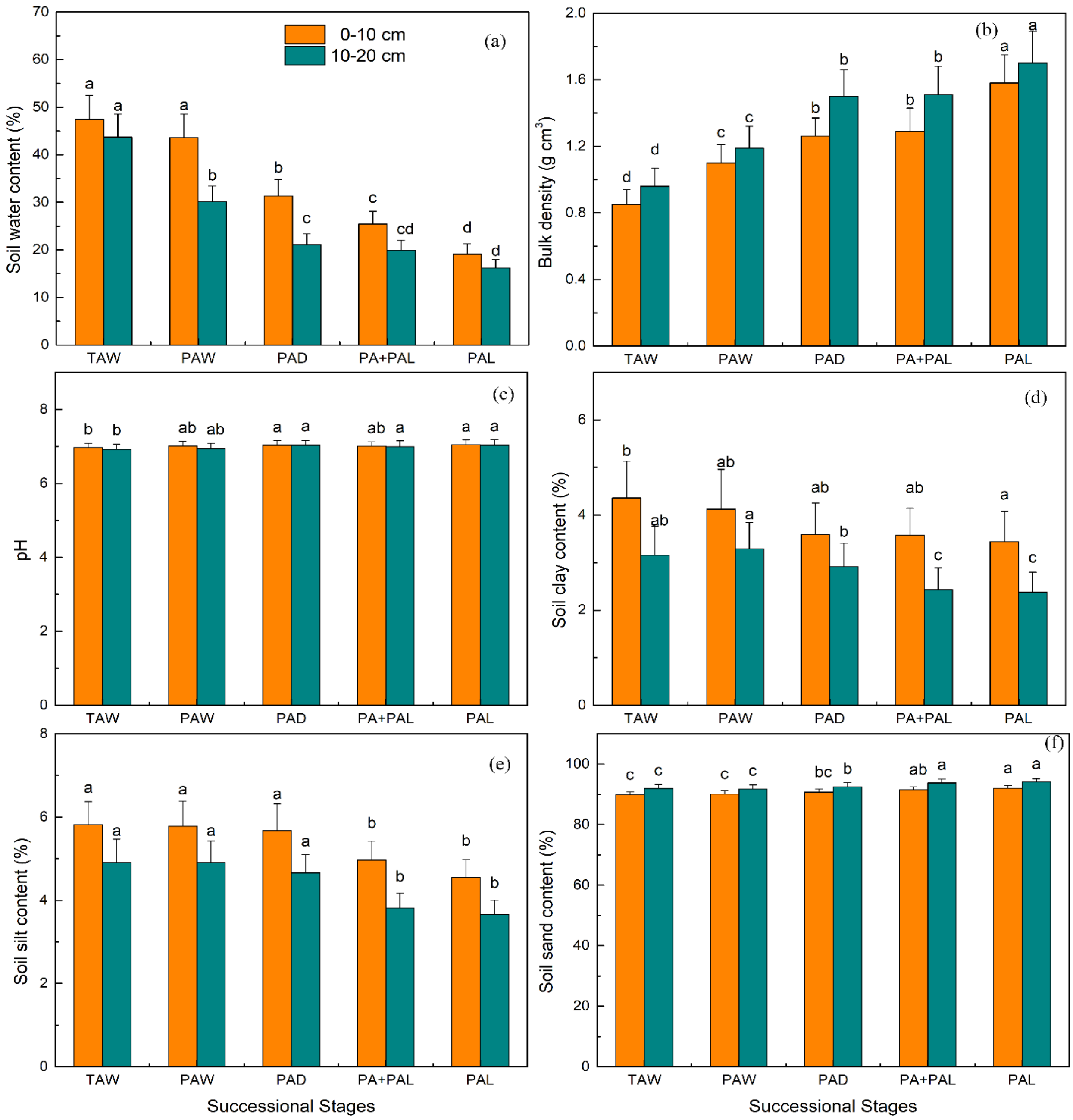

Figure S2. Soil water content (**a**), bulk density (**b**), pH (**c**), soil clay content (**d**), soil silt content (**e**) and soil sand content (**f**) in different stages of wetland drying process in October. Values are Mean ± SE (n = 9). Different letters over the bars indicate statistically significant differences between different stages. TAW, T. *augustifolia* wetland; PAW, P. *australis* wetland; PAD, P. *australis* dry land; PA+PAL, mixed grassland with P. *australis* and P. *arundinacea* L.; PAL, P. *arundinacea* L. grassland.

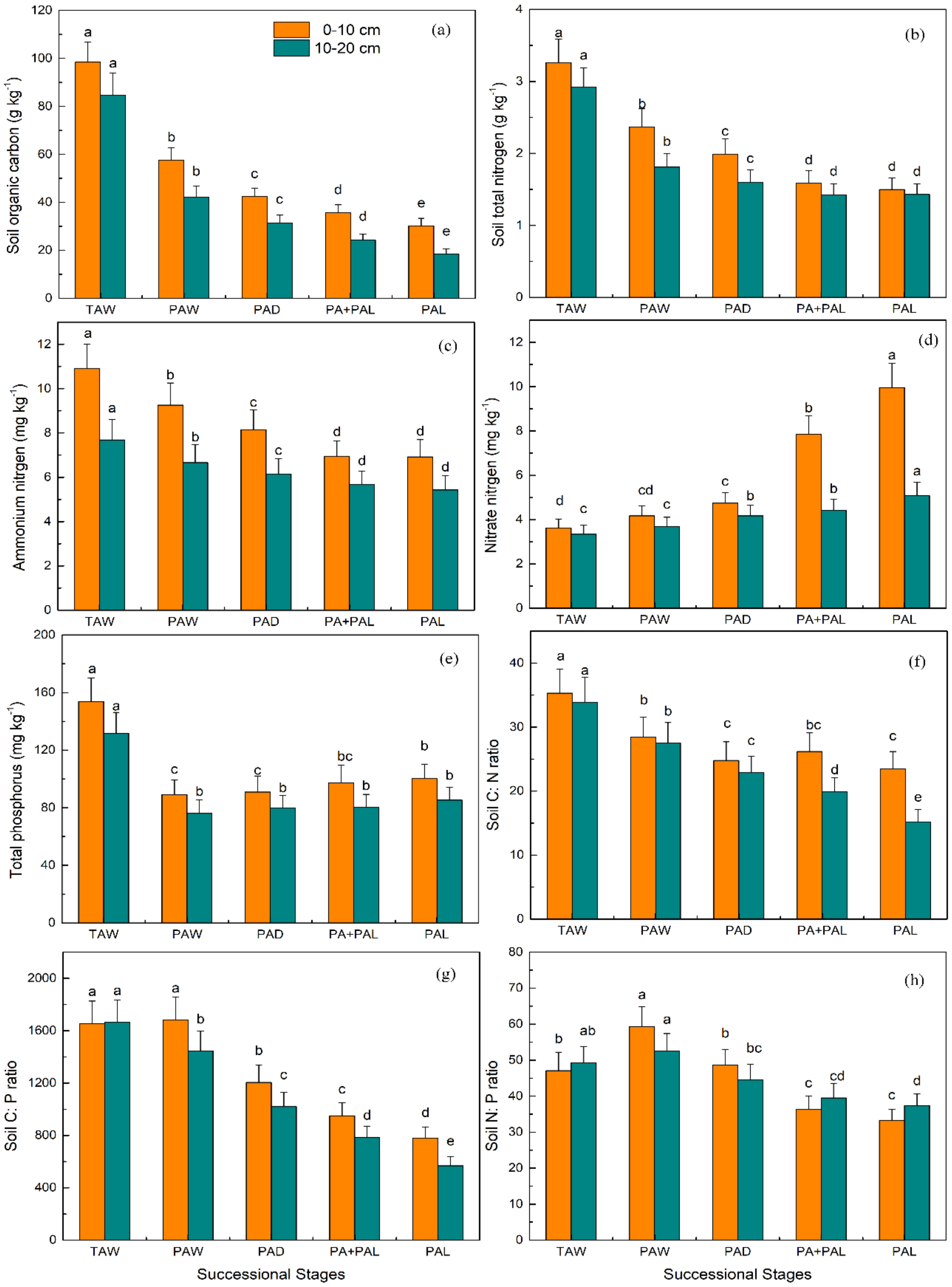

Figure S3. Soil organic carbon (**a**), total nitrogen content (**b**), ammonium nitrogen (**c**), nitrate nitrogen (**d**), total phosphorus content (**e**), soil C: N ratio (**f**), soil C: P ratio (**g**) and soil N: P ratio (**h**) in different stages of wetland drying process in June. Values are Mean ± SE (*n* = 9). Different letters over the bars indicate statistically significant differences between different stages. TAW, T. *augustifolia* wetland; PAW, P. *australis* wetland; PAD, P. *australis* dry land; PA+PAL, mixed grassland with P. *australis* and P. *arundinacea* L.; PAL, P. *arundinacea* L. grassland.

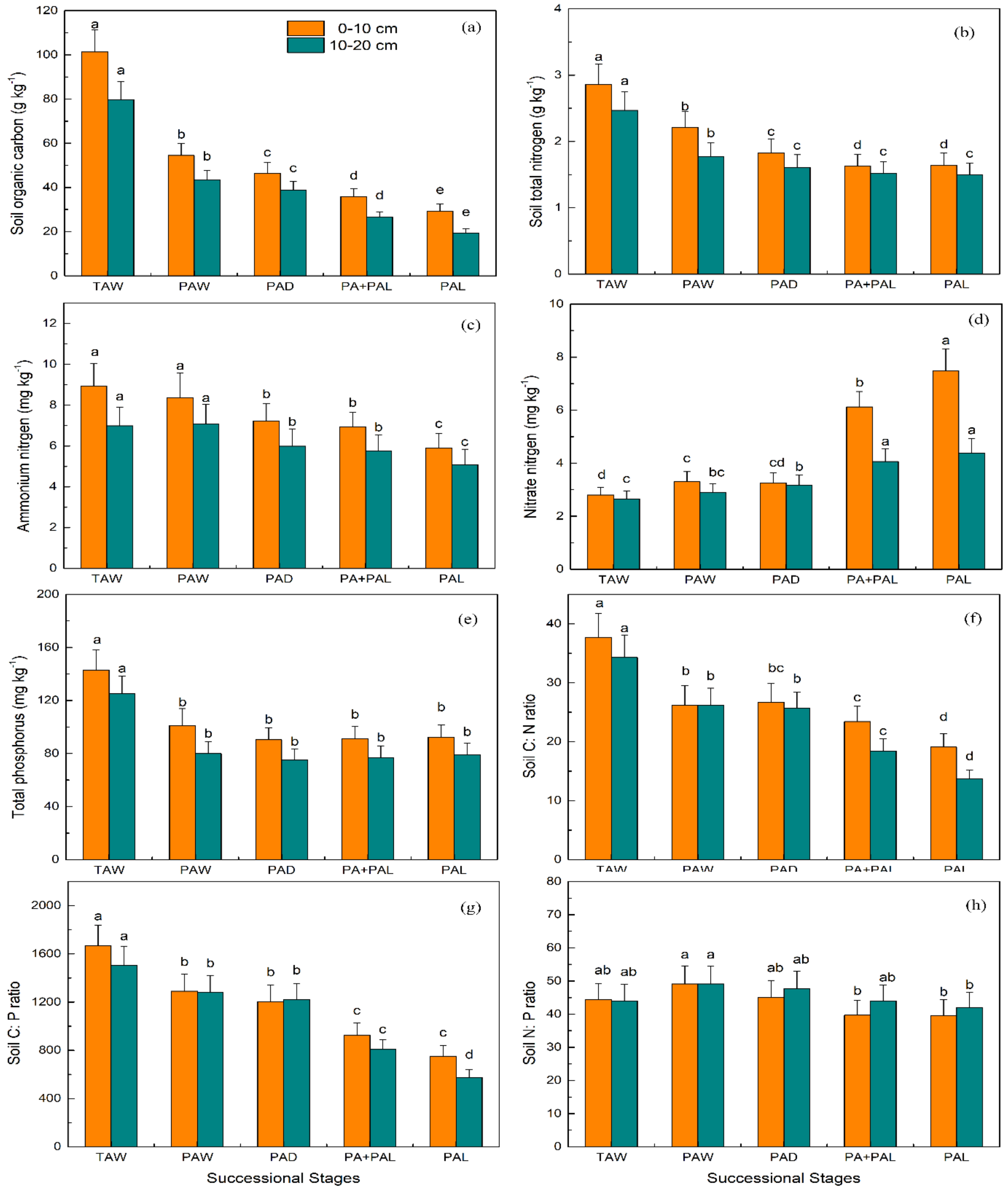

Figure S4. Soil organic carbon (**a**), total nitrogen content (**b**), ammonium nitrogen (**c**), nitrate nitrogen (**d**), total phosphorus content (**e**), soil C: N ratio (**f**), soil C: P ratio (**g**) and soil N: P ratio (**h**) in different stages of wetland drying process in October. Values are Mean ± SE (n = 9). Different letters over the bars indicate statistically significant differences between different stages. TAW, T. *augustifolia* wetland; PAW, P. *australis* wetland; PAD, P. *australis* dry land; PA+PAL, mixed grassland with P. *australis* and P. *arundinacea* L.; PAL, P. *arundinacea* L. grassland.

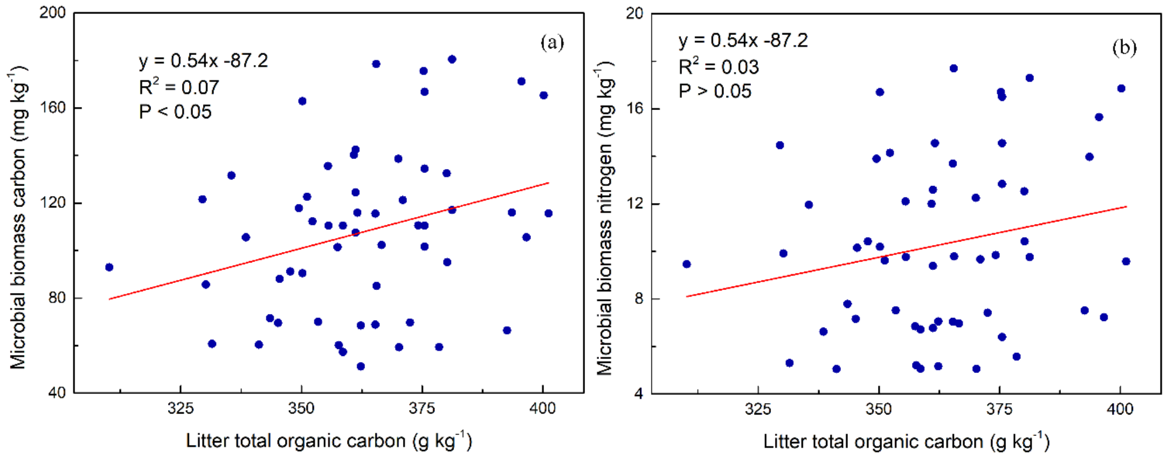

Figure S5. The relationships between soil MBC (**a**) and MBN (**b**) contents and litter total organic carbon content.

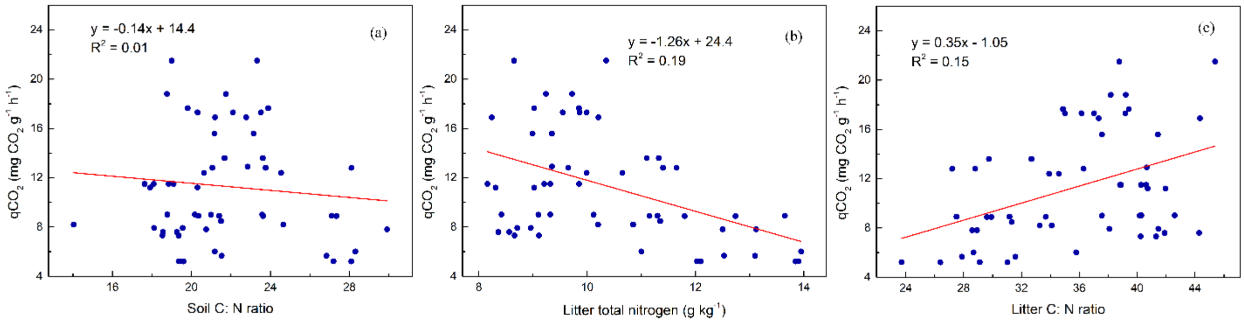

Figure S6. The relationships between soil microbial metabolic quotient (qCO_2) with soil C: N ratio (**a**), litter total nitrogen content (**b**) and litter C: N ratio (**c**).

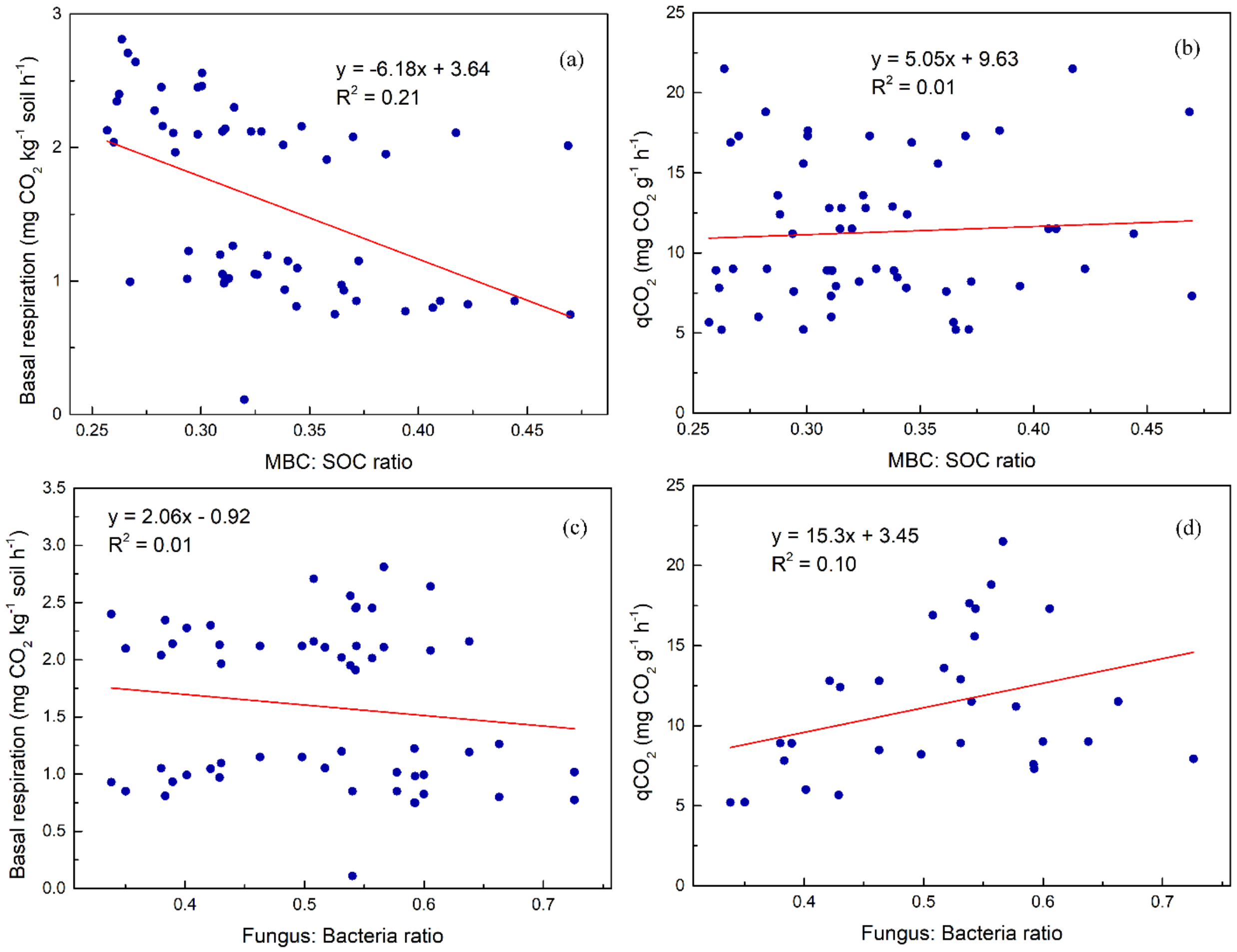

Figure S7. The relationships between soil basal respiration and microbial quotient (MBC: SOC ratio) (**a**) and fungi: bacteria ratio (**c**) and the relationships between soil microbial metabolic quotient (qCO_2) (**b**) and microbial quotient (MBC: SOC ratio) and fungi: bacteria ratio (**d**).
